# Surprise Lymph Node Histology in a Patient with Ovarian Serous Carcinoma

**DOI:** 10.1155/2020/6468124

**Published:** 2020-02-10

**Authors:** Jayanthi Karunanithi, Inny Busmanis

**Affiliations:** Department of Histopathology, Singapore General Hospital, Singapore

## Abstract

We report a case of a sixty-three-year-old Chinese female with a known past history of primary, biopsy-diagnosed, ovarian high-grade serous carcinoma. Following three cycles of chemotherapy, she underwent total hysterectomy, bilateral salpingo-oophorectomy cytoreductive surgery with lymphadenectomy, and multiple peritoneal biopsies. In this situation, the lymph nodes would be expected to demonstrate possible residual metastatic serous carcinoma with or without chemotherapy effect. The final diagnostic assessment in the lymph nodes, in this patient, however, was a rare combination of the following pathologies: metastatic serous carcinoma, with areas of chemotherapy effect, and incidental PEComatosis, focally in association with endometriosis, both within lymph nodes and surrounding connective tissue. PEComas have been described in patients with the tuberous sclerosis complex, but the current patient was not known to suffer from this condition. This case is also unusual, as although PEComas have been described as arising in the female genital tract, the associated phenomenon of endometriosis is exceedingly rare, and this is the first known reported case of lymph nodes harbouring a similar combination of pathologies.

## 1. Introduction

Perivascular epithelioid cell tumours (PEComas) represent a group of mesenchymal tumours that express both smooth muscle and melanocytic markers. They were first recognised as an entity in the gynaecological tract in the 2002 World Health Organization (WHO) classification of tumours of the female genital tract and have a predilection for a uterine location [1]. At present, the normal tissue counterpart of PEComas is largely unknown. With the increased practice of surgical lymph node dissections and debulking for gynaecological malignancies, incidental minute perivascular epithelioid cell (PEC) aggregates or tumours, especially in lymph nodes, have been increasingly reported in the literature. We present a case of incidental perivascular epithelioid cell collections (PEComatosis) in lymph nodes and the omentum with associated multiple pathologies.

## 2. Case Presentation

The patient is a sixty-three-year-old Chinese female with a past medical history of hypertension, hyperlipidemia, and hepatitis B. She was diagnosed with high-grade serous carcinoma of the ovary, following which she underwent three cycles of chemotherapy. Three months after completion of treatment, total hysterectomy, bilateral salpingo-oophorectomy cytoreductive surgery with pelvic lymph node dissection, and peritoneal biopsies were performed.

**Figure 1:**
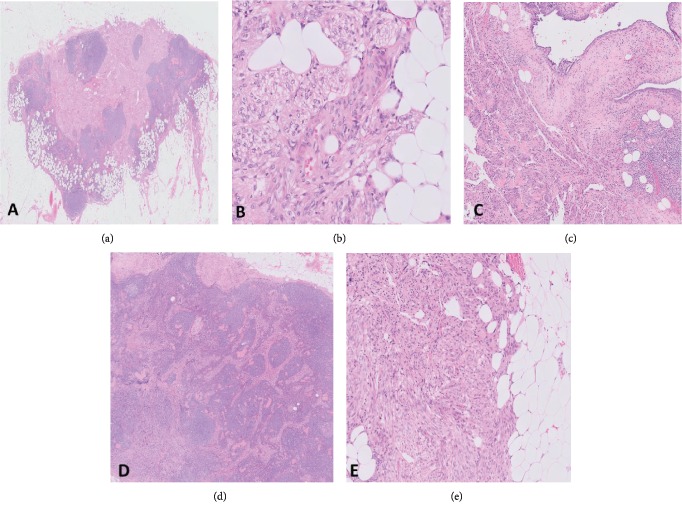
Histology showing (a) lymph node (H&E, 4x) involved by a lesion containing (b) fascicles resembling smooth muscle (H&E, 60x) with associated (c) endometriosis (H&E, 20x) and (d) adjacent foci of metastatic serous carcinoma (H&E, 20x). (e) Similar spindle cell bundles were also present in the perinodal adipose tissue (H&E, 40x).

Microscopic findings were residual high-grade serous carcinoma predominantly involving the left ovary and fallopian tube, with metastatic spread to multiple other adnexal sites, uterine and transverse colon serosa, and the omentum. Multiple lymph nodes examined also showed metastatic carcinoma as well as endometriosis with generally scant amount of endometrial type stroma. Other endometrial glands were surrounded by well-defined fascicles of spindle cells resembling smooth muscle. Aggregates of similar smooth muscle-like spindle cells were also seen in isolation within nodal tissue and perinodal adipose tissue, in the absence of any associated endometrial glands (Figure 1).

**Figure 2:**
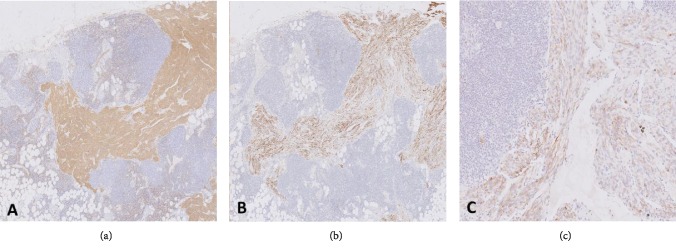
Positive immunohistochemistry of the spindle cell bundles with (a) desmin (10x), (b) SMA (10x), and (c) HMB45 (20x).

The spindle cells were immunoreactive for smooth muscle actin and desmin as well as focally positive with HMB45, confirming a perivascular epithelioid cell (PEC) immunophenotype (Figure 2). The final diagnostic assessment in the lymph nodes was therefore the rare combination of the following pathologies: metastatic serous carcinoma, with associated chemotherapy effect, and incidental PEComatosis, focally, but not invariably, in association with endometriosis, both within lymph nodes and surrounding connective tissue.

## 3. Discussion

PEComas are strongly associated with tuberous sclerosis complex [2], but in this case, the patient was not known to suffer from this condition. Similarly, Nagasaka et al. reported a lack of association of PEComas with tuberous sclerosis complex in their case series. The “PEC nests” described were small, ranging between 0.8 mm and 10 mm, and found incidentally during pelvic lymph node dissections for gynaecological malignancies [2], similar to our current case.

Lymph node involvement by either PEComatosis or endometriosis, although uncommon, has been reported [3]. However, to our knowledge, this is the first known case of concurrent lymph node involvement by these multiple pathologies. This poses a diagnostic pitfall.

While most reported PEComas have an epithelioid morphology, they can show a spectrum of cytological features from purely spindled to purely epithelioid as well as combinations of the two [2]. With the purely spindled morphology of the aggregates seen in this case, the differential diagnoses included smooth muscle metaplasia in association with endometriosis [2] as well as the phenomenon of leiomyomatosis within pelvic lymph nodes. Unless these differentials are considered, the adequate spectrum of immunohistochemical stains to distinguish between these entities would not be utilised. The cells of PEComatosis, smooth muscle metaplasia, and leiomyomatosis stain for smooth muscle markers such as desmin and smooth muscle actin (SMA), but only PEComatosis additionally shows a positive reaction with melanocytic markers.

PEComas are usually benign, but cases of malignant transformation have been reported [4]. The prognosis of incidentally found PEComatosis is not well established due to the insufficient number of cases with adequate follow-up reported in the current literature.

This case represents a unique constellation of features, but as PEComas can be seen in isolation in the absence of any associated malignancy or endometriosis, the relationship of the latter in PEComa pathogenesis remains conjectural.
